# *AKIRIN1*: A Potential New Reference Gene in Human Natural Killer Cells and Granulocytes in Sepsis

**DOI:** 10.3390/ijms20092290

**Published:** 2019-05-09

**Authors:** Anna Coulibaly, Sonia Y. Velásquez, Carsten Sticht, Ana Sofia Figueiredo, Bianca S. Himmelhan, Jutta Schulte, Timo Sturm, Franz-Simon Centner, Jochen J. Schöttler, Manfred Thiel, Holger A. Lindner

**Affiliations:** 1 Department of Anesthesiology and Surgical Intensive Care Medicine, University Medical Center Mannheim, Medical Faculty Mannheim, Heidelberg University, 68167 Mannheim, Germany; Anna.Coulibaly@medma.uni-heidelberg.de (A.C.); Sonia.Velasquez@medma.uni-heidelberg.de (S.Y.V.); sofia.figueiredo@bioquant.uni-heidelberg.de (A.S.F.); Bianca.Himmelhan@medma.uni-heidelberg.de (B.S.H.); Jutta.Schulte@medma.uni-heidelberg.de (J.S.); timo.sturm@med.uni-heidelberg.de (T.S.); franz-simon.centner@umm.de (F.-S.C.); jochen.schoettler@umm.de (J.J.S.); Manfred.Thiel@umm.de (M.T.); 2 Medical Research Center, Medical Faculty Mannheim, Heidelberg University, 68167 Mannheim, Germany; Carsten.Sticht@medma.uni-heidelberg.de

**Keywords:** *AKIRIN1*, granulocytes, natural killer cells, reference gene, sepsis, systemic inflammatory response syndrome

## Abstract

Timely and reliable distinction of sepsis from non-infectious systemic inflammatory response syndrome (SIRS) supports adequate antimicrobial therapy and saves lives but is clinically challenging. Blood transcriptional profiling promises to deliver insights into the pathomechanisms of SIRS and sepsis and to accelerate the discovery of urgently sought sepsis biomarkers. However, suitable reference genes for normalizing gene expression in these disease conditions are lacking. In addition, variability in blood leukocyte subtype composition complicates gene profile interpretation. Here, we aimed to identify potential reference genes in natural killer (NK) cells and granulocytes from patients with SIRS and sepsis on intensive care unit (ICU) admission. Discovery by a two-step probabilistic selection from microarray data followed by validation through branched DNA assays in independent patients revealed several candidate reference genes in NK cells including *AKIRIN1*, *PPP6R3*, *TAX1BP1*, and *ADRBK1*. Initially, no candidate genes could be validated in patient granulocytes. However, we determined highly similar *AKIRIN1* expression also in SIRS and sepsis granulocytes and no change by in vitro LPS challenge in granulocytes from healthy donors. Inspection of external neutrophil transcriptome datasets further support unchanged *AKIRIN1* expression in human systemic inflammation. As a potential new reference gene in NK cells and granulocytes in infectious and inflammatory diseases, *AKIRIN1* may improve our pathomechanistic understanding of SIRS and sepsis and help identifying new sepsis biomarkers.

## 1. Introduction

Systemic inflammatory response syndrome (SIRS) due to infection classically defines sepsis [1,2]. Additional organ dysfunction classically defines severe sepsis, and severe sepsis plus fluid refractory hypotension constitutes septic shock [1,2]. According to the more recent sepsis-3 definition, sepsis is defined as life-threatening organ dysfunction caused by a dysregulated host response to infection [3]. Timely antimicrobial therapy continues to be crucial to saving patient lives. Yet, due to the high prevalence of non-infectious SIRS and of organ dysfunction in these patients, early sepsis diagnosis is still a major clinical challenge [4,5,6]. Indicators that reliably distinguish between infectious and sterile inflammation, other than microbial detection, are lacking demonstrating an urgent need for a better understanding of the pathomechanisms of SIRS and sepsis and the identification of expeditious sepsis biomarkers [7,8]. Transcriptional profiling of whole blood leukocytes promises to reveal new insights into disease mechanisms and potential biomarkers of sepsis [9,10,11]. Progress towards this end is, however, still hampered by a lack of reference genes suitable to normalize blood leukocyte gene expression in sepsis and control patients. In addition, whole blood gene signatures are influenced by changes in both leukocyte gene expression patterns and the relative abundance of the different subtypes of leukocytes [12,13]. In conditions of acute systemic inflammation, emergency granulopoiesis governs white blood cell counts (WBCs) [14], and counts of lymphocytes and monocyte subsets rapidly undergo differential changes [15,16]. A deeper understanding of the disease processes that underlie sepsis may thus be gained, and new diagnostic gene signatures may be unveiled by targeted and leukocyte subtype specific gene expression analyses using suitable reference genes. 

In sepsis, granulocytes suffer dysfunctional migration and antimicrobial activity, restoration of which improved survival in mice [17,18]. Mouse NK cells exacerbate detrimental hyperinflammation in experimental sepsis, predominantly, through interleukin-15 (IL-15) induced interferon-γ production, but their dysfunction also appears to contribute to subsequent immunosuppression [19]. In human sepsis; however, the roles of granulocytes [20] and NK cells [19,21] for patient outcome remain ambiguous. 

In global transcriptome analyses, including blood transcriptomics in patients with sepsis and with SIRS as critically ill controls, statistical models have been used for the normalization of signal intensities and to allow comparison of gene expression levels between samples [10,11,22]. Yet, targeted validation and further analyses of differential gene expression in smaller numbers of selected genes relies on a suitable reference measure for the normalization of gene expression levels. The expression level of a given gene may refer to the quantity of RNA molecules transcribed from that gene and, on average, present per cell, i.e., its mean cellular abundance. Alternatively, expression levels may refer to cellular transcript concentrations. In targeted gene expression analyses, such as in the evaluation of a gene signature, cellular transcript abundance is commonly evaluated using cell number or the amount of input RNA as a reference for normalization [23].

However, identification and use of an endogenous gene with stable expression across comparison groups as a reference for expression normalization is strongly recommended as it accounts for technical variation during sample processing [24,25]. Yet, results from studies to identify such reference genes from transcriptome data [26] and to validate selected candidates by RT-PCR [27] have questioned the existence of a reference gene that is suitable to normalize gene expression universally across cell types, diseases, and experimental conditions. Instead, the use of the geometric mean of multiple genes selected for stable expression as a reference has been recommended [28]. As for blood leukocytes in particular, neutrophils in healthy donors were recently reported to display substantially higher variabilities in their DNA methylation and gene expression patterns than monocytes and T cells [29]. It thus appears that inherently high transcriptional variability has so far complicated identification of suitable reference genes in this cell type.

This study aimed to identify genes with invariant expression that therefore represent potential reference genes for future differential gene expression analysis in NK cells and granulocytes from patients with SIRS and sepsis.

## 2. Results

### 2.1. Patient Characteristics

We enrolled critically ill patients admitted to our surgical ICU with a recent diagnosis of post-traumatic SIRS or septic shock according to the 2001 International Sepsis Definitions Conference [2] in two different patient sets. For discovery, we collected blood from 20 presurgical, 19 SIRS, and 20 sepsis patients between August 2012 and April 2014 [30], and for validation from 22 SIRS and 23 sepsis patients between September 2016 and April 2017. In each round of enrollment, both NK cells and granulocytes were isolated from patient blood. Demographic and clinical characteristics were extracted from the hospital information system (Supplementary Table S1). Table 1 summarizes patient characteristics at ICU admission for discovery set cell preparations. Patient characteristics for NK cells and granulocytes are considered separately because not every cell preparation yielded sufficient cell purity as judged by flow cytometry or amount and integrity of RNA, i.e., not every preparation was eligible (see Materials and Methods). For both NK cells and granulocytes, the mean sequential organ failure assessment (SOFA) score was by almost 8 points higher in sepsis than in SIRS, and the C-reactive protein (CRP) mean concentration in blood plasma was about 3-fold higher. Contrarily, differences in WBCs for these two groups were not statistically significant. However, compared to presurgical patients, mean WBCs were 2-fold higher in sepsis and around 1.7-fold in SIRS. Mean blood lactate levels were significantly higher for sepsis than SIRS. 

Table 2 summarizes patient characteristics at ICU admission for eligible validation set NK cell and granulocyte preparations analogously to Table 1. Compared to the discovery set (Table 1), mean differences between SIRS and sepsis patients for SOFA scores were around 3.5 points lower and for CRP concentrations around 120 mg/mL higher. Higher values for both SOFA and CRP in sepsis remained statistically significant, while there were again no statistically significant differences in WBCs between the two groups. Validation set mean lactate levels were comparable to discovery set lactate levels for both SIRS NK cells and SIRS granulocytes (Table 1) but were significantly higher in sepsis than SIRS only for NK cells.

### 2.2. Reference Gene Discovery in Presurgical, SIRS, and Sepsis Patients

**Figure 1:**
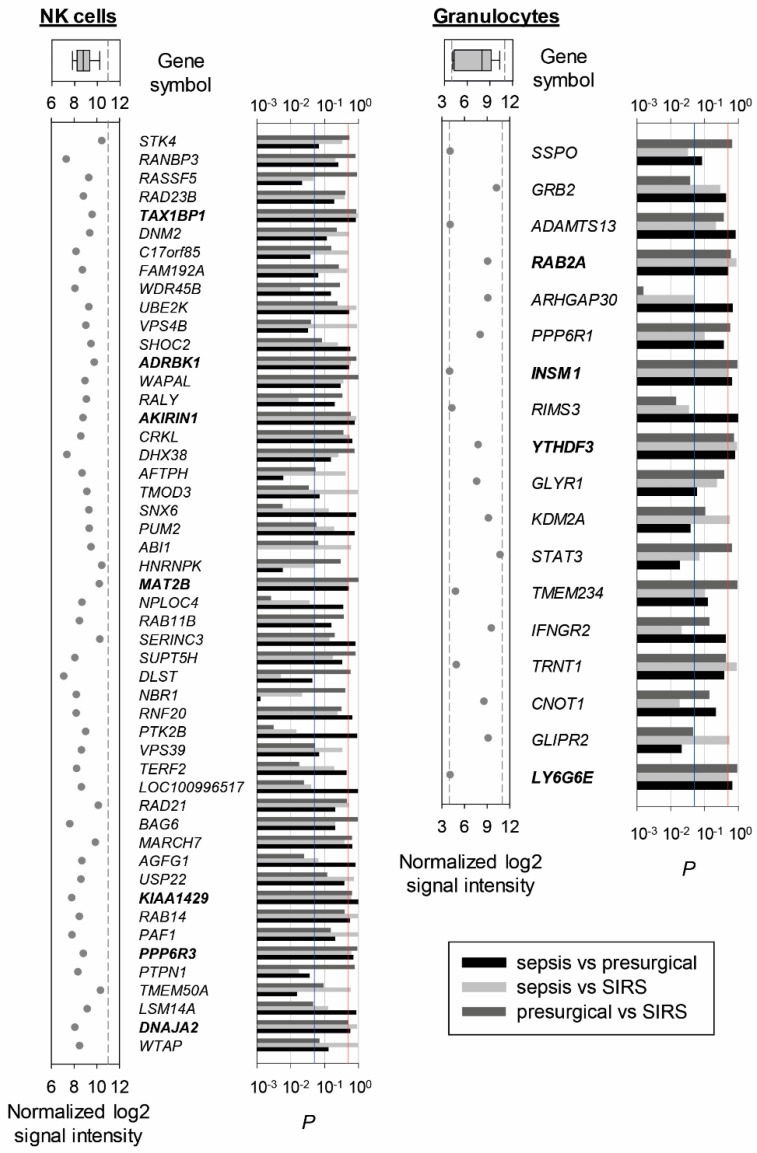
Discovery of candidate reference genes from microarray data of NK cells and granulocytes from presurgical, SIRS, and sepsis patients (discovery set). Genes without significant difference in abundance from one-way analysis of variance with false discovery rate correction (α > 0.05) and with intermediate normalized log_2_ signal intensity values were selected. They were ordered from top to bottom by decreasing variability across all 45 NK cell samples (19 presurgical, 16 SIRS, and 10 sepsis), of which the top 50 are shown on the left, and all 42 granulocyte samples (11 presurgical, 16 SIRS, and 15 sepsis) for which only the 18 genes shown on the right met the selection criteria. To the left of the gene symbols, normalized log_2_ signal intensities from the microarrays are given with dashed lines indicating the cutoff intensities from our selection criteria. On top, corresponding box plots with whiskers from 5^th^ to 95^th^ percentile and median are plotted. The bars to the right represent *p*-values from unadjusted *t*-tests for the corresponding three pairwise patient group comparisons. The blue line marks the significance threshold of *p* = 0.05, and the red line marks the threshold of *p* = 0.5 for selecting genes with putatively highly similar abundance among the three groups. Genes with above- or borderline-threshold *p*-values for all three comparisons are printed in bold.

**Figure 2:**
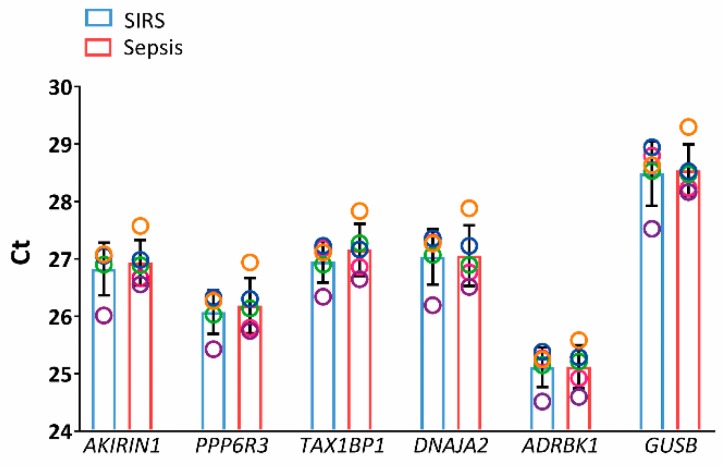
Gene expression analysis in NK cells from the discovery set by RT-PCR (SIRS: blue, sepsis: red). Candidate reference genes identified from microarray and additionally *GUSB* were tested in five randomly selected discovery set samples from each patient group. Threshold cycle (Ct) values are represented as mean values ± standard deviation (bars). For both groups, data are displayed as circles in a rainbow color scheme to identify values from same cell preparations, i.e., same patients within the respective group. *P*-values from the Wilcoxon rank-sum test were ≥ 0.69 throughout.

Out of the approximately 25,000 genes on the microarray, 0.53% in NK cells and 28.3% in granulocytes showed a significant difference in abundance in any of the pairwise comparisons of the three patient groups in the discovery set and were thus excluded. Among the 50 genes with the lowest global variability and intermediate signal intensities in NK cells (Figure 1, left), the standard deviation (sd) of the normalized log_2_ signal intensities ranged from 0.117 to 0.143. We sought to identify five candidate reference genes and selected *TAX1BP1*, *ADRBK1*, *AKIRIN1*, *KIAA1429*, *PPP6R3*, *MAT2B*, and *DNAJA2* as those with putatively equal transcript abundance in all three patient groups as judged from unadjusted *t*-tests. We excluded *KIAA1429* for further examination because no function or gene product had been described at the time of analysis, and we considered *MAT2B* and *DNAJA2* instead as potential fifth candidates. Both had one borderline below-threshold *p*-value out of three. We chose *DNAJA2* because only here both remaining *p*-values were clearly above-threshold. Invariant expression of our five candidate genes selected for NK cells and additionally of the known endogenous reference gene *GUSB* was confirmed in a subset of SIRS and sepsis samples from the discovery set by RT-PCR (Figure 2). 

**Figure 3:**
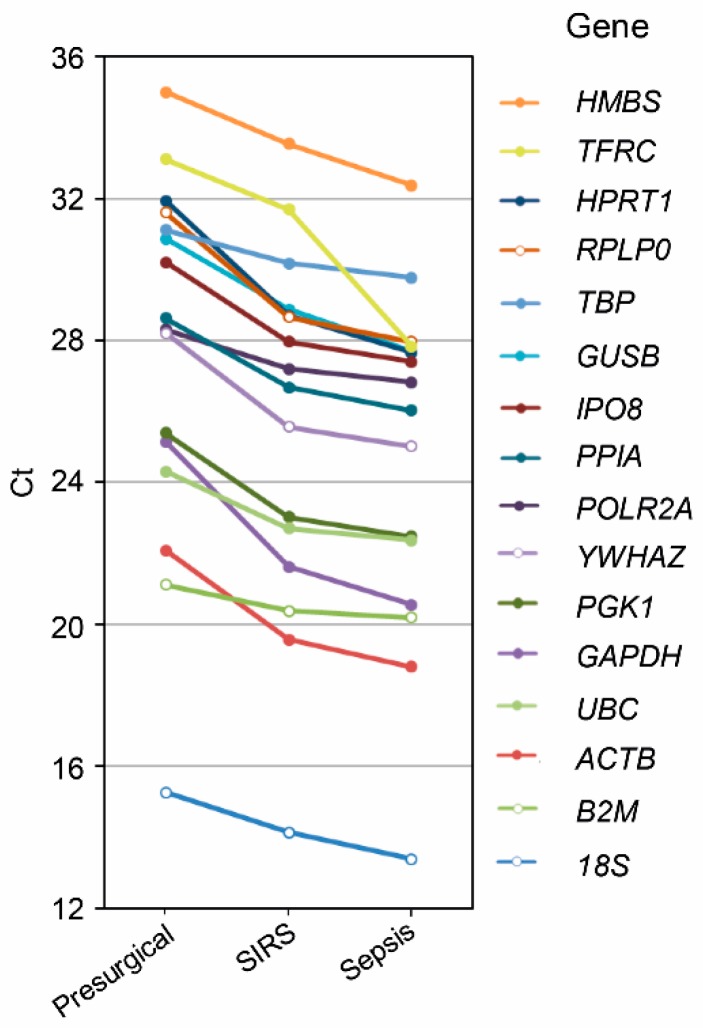
RT-PCR screen for endogenous reference genes in presurgical, SIRS, and sepsis discovery set granulocytes. Sixteen known reference genes assembled in the TaqMan Array Human Endogenous Control Panel were tested in randomly selected discovery set samples (presurgical, *n* = 5; SIRS, *n* = 5; sepsis, *n* = 6). Threshold cycle (Ct) values are represented as group averages. Data points are connected by lines to aid visual comparisons between groups. The overall mean coefficient of variation (CV) for these determinations was 4.3%. CV values were < 7.2% except for determinations of *18S* in SIRS (22.3%) and sepsis (14.4%).

Among the 18 low-variability genes shortlisted for granulocytes (Figure 1, right), the standard deviation of the normalized log_2_ signal intensities ranged from 0.111 to 0.144. Only four of them met our *t*-test-based selection criterion, namely, *RAB2*, *INSM1*, and *LY6G6E* that each had one borderline and two clear above-threshold *p*-values, and *YTHDF3* with all three *p*-values clearly above-threshold. We additionally screened 16 known endogenous reference genes in a subset of presurgical, SIRS, and sepsis granulocyte samples from the discovery set by RT-PCR (Figure 3). Expression levels were consistently higher for the SIRS compared to the presurgical patients (on average 4.7-fold) and for the sepsis compared to the SIRS patients (on average 2.4-fold). 

### 2.3. Validation in SIRS and Sepsis Patients

**Figure 4:**
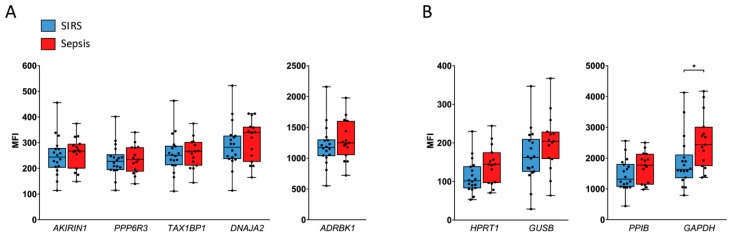
Comparison of candidate reference gene expression in NK cells from the validation set by QGP. Results for candidate reference genes identified from microarray (**A**) and four additional known reference genes (**B**) are grouped by similar signal intensity (MFI) ranges. Data are represented as blue (SIRS, *n* = 18) and red (sepsis, *n* = 15) box plots with whiskers from minimum to maximum, median, and dispersion plots. * *p* < 0.05 from the Wilcoxon rank-sum test.

We validated our candidate reference genes identified from microarray data in additional ICU patients with SIRS and sepsis on admission by QuantiGene Plex (QGP) assays. This analysis yielded highly similar expression levels for four of our five candidate reference genes in SIRS and sepsis NK cells, namely, *AKIRIN1*, *PPP6R3*, *TAX1BP1*, and *ADRBK1* (Figure 4A). On average, median fluorescence intensity (MFI) values were around 1.2-fold higher in sepsis compared to SIRS for *DNAJA2* (Figure 4A) and the known reference genes *HPRT1*, *GUSB*, and *PPIB* (Figure 4B), although these differences did not reach statistical significance. For *GAPDH*, an apparent 1.4-fold higher mean abundance in sepsis than SIRS; however, passed the threshold of significance (Figure 4B).

**Figure 5:**
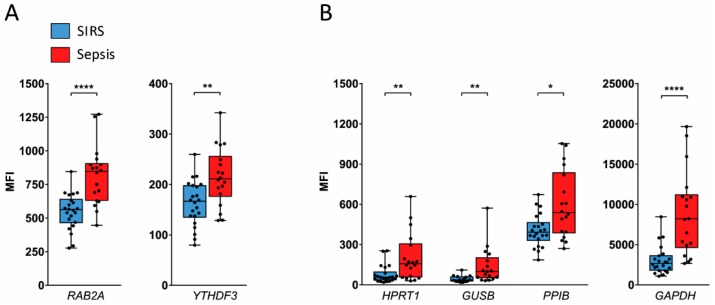
Comparison of candidate reference gene expression in granulocytes from the validation set by QGP. Results for two detectable candidate reference genes identified from microarray (**A**) and four additional known reference genes (**B**) are grouped by similar signal intensity (MFI) ranges. Data are represented as blue (SIRS, *n* = 22) and red (sepsis, *n* = 18) box plots with whiskers from minimum to maximum, median, and dispersion plots. As an exception, only 15 samples from patients with SIRS and 17 with sepsis were available for *GUSB*. * *p* < 0.05, ** *p* < 0.01, **** *p* < 0.0001 from the Wilcoxon rank-sum test.

In validation set granulocytes, expression of *INSM1* was undetectable by QGP, and only half of the samples yielded a signal above the limit of detection for *LY6G6E*. The other two candidate reference genes, *RAB2A* and *YTHDF3*, showed significant 1.5- and 1.3-fold higher mean expression levels, respectively, in sepsis than in SIRS (Figure 5A), as did the known reference genes *HPRT1* (2.6-fold), *GUSB* (3.2-fold), *PPIB* (1.5-fold), and *GAPDH* (2.9-fold) (Figure 5B). 

**Figure 6:**
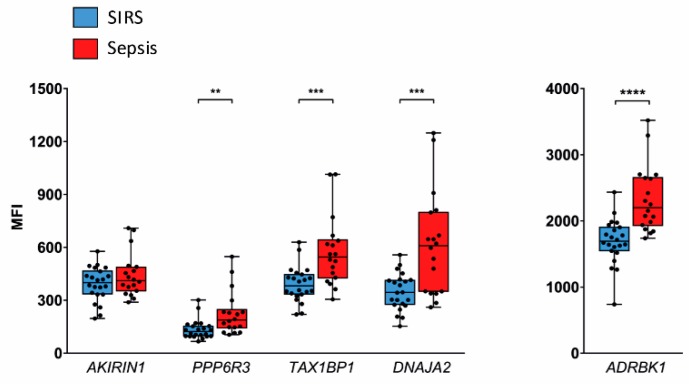
Assessment of candidate reference genes validated for NK cells in granulocytes from the validation set by QGP. Results are grouped by similar signal intensity (MFI) ranges. Data are represented as blue (SIRS, *n* = 22) and red (sepsis, *n* = 18) box plots with whiskers from minimum to maximum, median, and dispersion plots. ** *p* < 0.01, *** *p* < 0,001, **** *p* < 0.0001 from the Wilcoxon rank-sum test.

We additionally assessed the five candidate genes discovered in NK cells (Figure 4A) also in validation set granulocytes by QGP (Figure 6). In four of them, the mean abundance was again higher in sepsis than in SIRS, namely, *PPP6R3* (1.7-fold), *TAX1BP1* (1.5-fold), *DNAJA2* (1.8-fold), and *ADRBK1* (1.4-fold). However, *AKIRIN1* levels were highly similar in both patient groups.

### 2.4. AKIRIN1 Expression in External Neutrophil Transcriptome Datasets

Microarray data from our discovery set suggested approximately 20% lower *AKIRIN1* expression levels in both presurgical and SIRS granulocytes each compared to sepsis. We thus additionally considered *AKIRIN1* expression in external human peripheral blood neutrophil transcriptome datasets. Four curated microarray datasets were identified for an assessment. We calculated mean fold differences and *p*-values for pairwise comparisons of study groups as measures of group differences for each dataset as summarized in Table 3.

In two studies, Tang et al. (2007, 2008) recruited patients on ICU admission [31,32]. In the first study, patients with and without sepsis were enrolled in two separate phases to obtain training and validation set neutrophils [31]. In the second study, neutrophils from patients without sepsis and with confirmed Gram-positive, Gram-negative, or mixed sepsis were analyzed [32]. In both studies, *AKIRIN1* expression levels apparently did not differ between patient groups.

Silva et al. (2007) enrolled ICU patients with sepsis-induced acute lung injury (ALI) [33]. Neutrophils from same patients were cultured for 60 min in the presence of 1000 ng/mL high mobility group box 1 protein (HMGB1), 100 ng/mL lipopolysaccharide (LPS), and no additive (control). No changes in *AKIRIN1* expression levels are apparent from the available data.

Coldren et al. (2006) exposed healthy volunteers to LPS by bronchoscopic instillation [34]. Neutrophils from blood were analyzed both pre- and 16 h post-LPS treatment as well as neutrophils from bronchoalveolar lavage 16 h post LPS. In contrast to blood, lavage neutrophils were isolated by a negative selection method. In addition, circulating neutrophils were subjected to in vitro treatment with 100 ng/mL LPS for 60 min. Neither in vitro nor in vivo LPS challenge apparently altered *AKIRIN1* expression levels in the respective system itself. In vitro culture per se, however, appears to have increased *AKIRIN1* levels more than twofold compared to both circulating and alveolar neutrophils regardless of LPS challenge.

### 2.5. In Vitro Proinflammatory Stimulation of Healthy Donor Granulocytes

**Figure 7:**
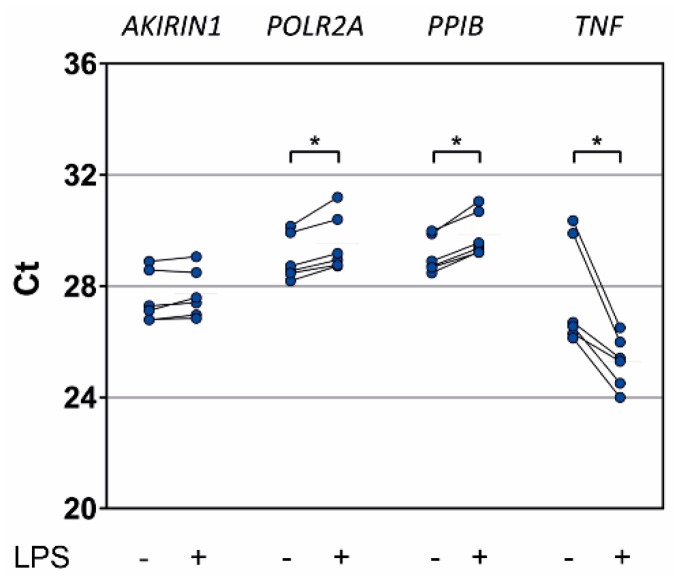
Reference and control gene expression in in vitro stimulated healthy donor granulocytes by RT-PCR. Granulocytes were isolated from whole blood of six healthy donors and cultured for 2 h with or without LPS (100 ng/mL). Data are represented as Ct values, and data points from same samples are connected by a line. * *p* < 0.05 from the Wilcoxon signed-rank test.

Similar to Silva et al. (2007) [33] and Coldren et al. (2006) [34], we used short-term LPS treatment as an in vitro inflammatory stimulus to probe changes in *AKIRIN1* expression in granulocytes from healthy donors (Figure 7). The expected increase of TNF-α gene (*TNF*) expression through LPS averaged 7.1-fold. Mean *POLR2A* and *PPIB* expression levels decreased 0.7- and 0.6-fold, respectively. Only *AKIRIN1* expression levels appeared almost unchanged (0.9-fold mean decrease, range 0.7- to 1.1-fold).

## 3. Discussion

The major observation of this study was that expression levels of *AKIRIN1* determined in NK cells and granulocytes isolated from peripheral blood were highly similar in patients admitted to the ICU with SIRS compared to sepsis. We introduce *AKIRIN1* as the first potential reference gene for future differential gene expression analysis in NK cells and granulocytes from critically ill patients with SIRS and sepsis. Moreover, LPS stimulated granulocytes from healthy donors showed invariant *AKIRIN1* expression. Despite many years of genome-wide transcriptional profiling in whole blood leukocytes from sepsis patients [35], suitable reference genes to normalize gene expression in sepsis and control patient whole blood for targeted analyses have so far eluded identification and, ultimately, may not exist. Recent whole blood gene expression studies to validate sepsis signatures [36] and to classify sepsis patients according to the immune response [37,38,39,40,41,42], to etiology [43], and to mortality as outcome [44,45] all relied on global transcriptome normalization without further targeted confirmation of differential expression in individual genes. Likewise, a recent microarray study of circulating neutrophils from ICU patients after the onset of septic shock compared to healthy controls also exclusively relied on global transcriptome normalization [46].

Differential gene expression of specific peripheral blood leukocyte subtypes considered individually can be expected to reveal a more consistent transcriptional response to sepsis than apparent in whole blood. Specific reference genes may thus be verifiable and facilitate future targeted validation of subtype specific gene expression differences between SIRS and sepsis and according gene function analyses. In our effort to discover invariant genes that could serve as such reference genes, selectively, in NK cells and granulocytes from SIRS and sepsis patients on ICU admission, we also included presurgical patients as a comparison group of hospitalized patients of similar age. This aimed to increase the probability of finding reference genes also applicable to noncritical conditions, which was, however, not further validated in this study, where the focus was on ICU patients. On average, these had approximately twofold higher WBCs than presurgical patients (Table 1), and SOFA scores were around twofold and CRP concentrations around fourfold higher in sepsis compared to SIRS (Table 1 and Table 2). This underscores stark contrasts between our patient groups in the degree of severity of illness (SOFA) and systemic inflammation (WBC and CRP). Our sepsis group was enrolled according to the sepsis-1/2 definition for septic shock [2]. All patients in this group also had sepsis according to sepsis-3. Our patient group assignments to SIRS and sepsis thus remain unaffected by the sepsis-3 definition. More than half of the sepsis patients for the discovery set samples (Table 1) and a little less than half for the validation set samples (Table 2) had lactate levels above 2 mM, and hence also qualified as septic shock according to sepsis-3 [3].

From the respective microarray experiment in NK cells and granulocytes from the discovery set, we first selected among putatively nondiscriminatory genes those with low global variability to favor interindividual similarity across presurgical, SIRS, and sepsis patient groups. A quality assessment of the microarray data is provided as Supplementary results. In a second selection step, we compared these patient groups for differential gene expression in each cell type with a more progressive statistical test than applied in the original microarray analysis (Figure 1). Our resultant selection of five candidate reference genes in NK cells, *AKIRIN1*, *PPP6R3*, *TAX1BP1*, *DNAJA2*, and *ADRBK1*, was initially confirmed by RT-PCR in a subset of the same samples (Figure 2). In selecting these specific genes, we also focused on candidates with known gene function. Strictly, a known function is not a requirement for suitability as a reference in expression normalization. However, knowing a gene’s biological role potentially indicates cellular conditions under which its expression may not be expected to remain stable and, therefore, represents an advantage. Genes such as *KIAA1429* and *MAT2B* still represent interesting candidates for future evaluation.

Compared to 4831 genes in NK cells, merely 18 genes in granulocytes remained after the first selection step, severely limiting the pool of potential candidates for the second step (Figure 1). *RAB2*, *INSM1*, *LY6G6E*, and *YTHDF3* were eventually judged to qualify as candidates. Because of the extremely poor quota of only 18 potential candidate genes, we additionally rescreened expression of 16 known reference genes in a subset of the same granulocyte samples by RT-PCR (Figure 3). For all these genes, we found a consistent increase in abundance from presurgical to SIRS to sepsis. Three of them were previously used to normalize RT-PCR data from human neutrophils in conditions of systemic inflammation. *18S*, *GAPDH*, and *ACTB* served as reference genes to compare sepsis and controls [31], *18S* and *GAPDH* to compare elderly and young sepsis patients [47], and *18S* to follow gene expression after LPS infusion [48]. In our RT-PCR screen (Figure 3), *18S* showed a 1–5 orders of magnitude higher mean expression level than the other 15 genes and suffered high variability in SIRS and sepsis which are both unfavorable features for a reference gene. *GAPDH* and *ACTB* expression profiles determined by RT-PCR were highly similar (Figure 3), and higher *GAPDH* expression in sepsis compared to SIRS was confirmed by QGP in this study (Figure 5B) and previously in sepsis compared to age- and sex-matched outpatients by Cummings et al. (2014) [49]. Together, these results challenge the suitability of *18S*, *GAPDH*, and *ACTB* as reference genes in sepsis granulocytes.

To validate our candidate reference genes in SIRS and sepsis, we conducted a second round of patient enrolment, performed isolation of NK cells and granulocytes, and used an alternative assay principle, i.e., QGP, to assess gene expression and, thereby, avoid exclusive dependency of our results on microarray screening technology. Compared to the discovery set (Table 1), validation set patients showed a lower group contrast in disease severity as judged by SOFA scores and a higher contrast in systemic inflammation as judged by plasma CRP concentrations (Table 2). Both characteristics were still much worse in sepsis than in SIRS. QGP assays in NK cells from the validation set confirmed equal expression levels in both patient groups for our candidate reference genes *AKIRIN1*, *PPP6R3*, *TAX1BP1*, and *ADRBK1* (Figure 4). Yet, QGP validation in granulocytes failed to confirm any of our four candidates and the four known reference genes. Expression levels were consistently higher in sepsis than in SIRS (Figure 5). In addition, we observed the same trend for our original NK cell candidate genes in validation set granulocytes except for *AKIRIN1*, which showed highly similar expression levels in granulocytes of both patient groups (Figure 6).

An assessment of differential expression in four external transcriptome data sets further supports unchanged *AKIRIN1* expression in human peripheral blood neutrophils in inflammation (Table 3). No differences in *AKIRIN1* levels were apparent between control and sepsis on ICU admission [31,32] as well as between healthy volunteers before and after lung exposure to LPS and, additionally, compared to neutrophils sequestered in the LPS-instilled lungs [34]. Likewise, in vitro LPS treatment altered *AKIRIN1* expression in neutrophils neither from patients with sepsis-induced ALI [33] nor from healthy volunteers [34]. Notably, in vitro culture of neutrophils itself increased *AKIRIN1* abundance slightly above twofold [34], possibly reflecting a rapid set point adjustment.

We sought to validate unchanged *AKIRIN1* expression in granulocytes from healthy donors after in vitro exposure to LPS as observed in two of the external datasets (Table 3) [33,34]. For comparison, we included *POLR2A* (Figure 3) and *PPIB* [50] due to their relatively high stabilities among known reference genes with intermediate expression levels. In granulocytes from healthy donors, both genes were; however, downregulated by LPS while *AKIRIN1* indeed appears to be a suitable reference gene also for in vitro LPS challenge (Figure 7).

*AKIRIN1* encodes a ubiquitously expressed nuclear protein of 192 amino acids in length and is promyeogenic in mice [51]. *AKIRIN1* knockout in mice showed no obvious phenotype, while its paralog *AKIRIN2* [52] was required for embryonic development [53]. As its orthologue in *Drosophila* and *Caenorhabditis*, the akirin-2 protein mediates innate immune responses by bridging chromatin-remodelers and transcription factors including NF-κB [54,55]. Details on the molecular interactions of akirin-1, however, have not yet been described, and it is not known whether it also plays a role in immunity. It is tempting to speculate that *AKIRIN1* fulfills a maintenance function in human granulocytes and thus may merit the description “housekeeping gene”. In accordance with the “piggyback” hypothesis to explain stable conservation of redundant genes [56], a putative function of *AKIRIN1* in innate immune cells may have been coselected with its promyogenic activity or other functions. This gives further rise to the question whether *AKIRIN1* is also stably expressed in murine granulocytes.

Genes with low expression variabilities in specific cell types, tissues, and patient populations have previously been characterized by high connectivities in coexpression and protein–protein interaction network analyses [57,58,59]. They tend to be functionally and physically localized to the center of signaling pathways including the nucleus [57], where akirin-1 and -2 were also detected [51]. It remains to be seen whether, like akirin-2, akirin-1 engages in gene regulation.

It has to be considered that our comparisons of gene expression data from RT-PCR depended on reliable RNA determinations and assumed stable mRNA-to-rRNA ratios [24], which has not yet been verified in SIRS and sepsis granulocytes. Contrarily, comparisons of our QGP data were based on cell counts which may be affected by cellular integrity. However, we observed no patient group differences in cell viabilities and thus cellular integrities. Importantly, normalization of RT-PCR and QGP data using a suitable endogenous reference gene not only accounts for technical variation. By turning either assay readout into a gene ratio, the results also become insensitive to differences in the cellular rRNA fraction and overall cellular transcript concentration.

In conclusion, we propose to include *AKIRIN1* in future screens of canonical reference genes for normalizing gene expression in peripheral blood NK cells and granulocytes in patients with SIRS and sepsis. We also put forward *PPP6R3*, *TAX1BP1*, and more highly expressed *ADRBK1* as new reference genes in SIRS and sepsis NK cells. By comparison, granulocytes showed much larger differences in global gene expression between patient groups. Here, we demonstrated highly similar *AKIRIN1* expression levels in both NK cells and granulocytes from patients with SIRS and sepsis at ICU admission as well as in granulocytes from healthy donors after in vitro LPS challenge. The identification of potential endogenous reference genes in NK cells and granulocytes from patients with inflammatory and infectious conditions may spur targeted transcriptional analyses for the discovery of NK cell and granulocyte-based biomarkers and provide a better understanding of the pathophysiological roles of these cell types. It will also be interesting to see whether and how the stable expression of *AKIRIN1* is related to its protein function.

## 4. Materials and Methods 

### 4.1. Participants

This study was conducted at the Department of Anesthesiology and Surgical Intensive Care Medicine at the University Medical Center Mannheim. It was reviewed by the Medical Ethics Commission II of the Medical Faculty Mannheim, Heidelberg University. Approval for this research was obtained from this committee (2011-411M-MA, 11.11.2011; 2016-521N-MA, 16.03.2016) under the condition that it was conducted ethically in accordance with the World Medical Association Declaration of Helsinki. Informed consent to participate in the study was obtained from all participants or their legal guardian in the case of critically ill patients unable to consent. All participants were aged ≥ 18 years. Blood of critically ill patients with a recent diagnosis of posttraumatic SIRS or septic shock according to the 2001 International Sepsis Definitions Conference [2] was collected within 24 h after ICU admission and was subjected to isolation of both NK cells and granulocytes. The SOFA score (range 0–24) [60] was determined on admission. Patients waiting for elective surgery were recruited on presurgical examination. Exclusion criteria were pregnancy, cardiopulmonary resuscitation, glucocorticoid therapy, end-stage renal disease, liver disease, and previous organ transplantation. Blood for in vitro cell stimulations was obtained from healthy volunteers. 

### 4.2. Cell Isolation and Stimulation

Blood was collected in S-Monovette® K3E tubes (Sarstedt, Nümbrecht, Germany) by venipuncture with presurgical patients and healthy donors and from a central venous catheter with ICU patients. Whole blood was subjected to immunomagnetic cell separation (MACS®, Miltenyi Biotec, Bergisch Gladbach, Germany). For patient NK cell isolation, peripheral blood mononuclear cells (PBMCs) were extracted from 32 mL blood by a Ficoll-Paque gradient (GE Healthcare Life Sciences, Glattbrugg, Switzerland). We subjected PBMCs to depletion using a labeling cocktail composed of CD15, CD14, CD3, and CD19 MicroBeads on an LD column and subsequent enrichment with CD56 MicroBeads on an MS column (Miltenyi Biotec). On average, eligible NK cell preparations stained 96.2 ± 4.6% (standard deviation) for CD56 and <1% each for CD15, CD14, CD3, and CD19 in flow cytometric analysis. Neutrophils and eosinophils, here collectively referred to as granulocytes, from patients and healthy donors were enriched from 2 mL blood by positive selection (StraightFrom™ Whole Blood CD15 MicroBeads, Miltenyi Biotec). On average, eligible granulocyte preparations stained 98.1 ± 6.0% for CD15 and 0.5 % ± 1.5% for CD14. Cells were counted and viabilities were determined by trypan blue staining using a CountessTM II automated cell counter (Invitrogen, Thermo Fisher, Waltham, United States). The overall mean viability of freshly prepared cells was 94.3 ± 8.6% and did not differ between patient groups.

Granulocytes from healthy donors were plated at 10^6^/mL in RPMI 1640 medium (Sigma, Munich, Germany) supplemented with 10% fetal bovine serum (FBS) and 2 mM L-glutamine and were maintained in a cell culture incubator with 5% CO_2_. Cells were treated for 2 h with 100 ng/mL LPS (Sigma) in phosphate buffered saline (PBS) using an equal volume of PBS as a control.

### 4.3. Flow Cytometry

Proportions of granulocytes (CD15+), NK cells (CD3-CD56+), monocytes (CD14+), T cells (CD3+) and B cells (CD19+) were assessed in MACS isolated cells by multicolor flow cytometry on a FACSCanto II cytometer (BD Biosciences, San Jose, CA, USA). FlowJo V10 (Tree Star, Ashland, OR, United States) software was used for analysis. We acquired at least 10^4^ events each for MACS isolated NK cells and granulocytes. Debris and aggregates were excluded in a forward scatter height versus area plot. Sequential gates on biparametric dot plots were used to calculate the percentages of the different leukocyte populations. Details on monoclonal antibody-fluorochrome conjugates (BD Biosciences) and staining panels are given in Supplementary Table S2.

### 4.4. Total RNA

Total RNA for microarray and RT-PCR was purified from isolated discovery set cells and in vitro stimulated cells stored in RNAlater (Ambion®, Thermo Fisher). For granulocytes, we used the miRVanaTM miRNA isolation kit (Applied Biosystems, Thermo Fisher). For NK cells, we employed a reported modification thereof [61]. Isolated RNA was DNase-treated (DNA-free kit, Ambion) and spectrometrically quantified (Tecan Infinite® M200 NanoQuant, Tecan, Männedorf, Switzerland). Eligible preparations yielded RNA concentrations of on average 220 ng/µL with RNA integrity numbers consistently > 8 (Agilent Bioanalyzer 2100, Agilent, Santa Clara, United States).

### 4.5. Microarray and Selection of Candidate Endogenous Reference Genes

Hugene-2_0-st-type arrays (Affymetrix, Thermo Fisher, Santa Clara, CA, United States) were run on an Affymetrix GeneChipTM platform (Affymetrix Core Facility, Medical Research Center Mannheim) using a Custom CDF Version 18 for NK cells and Version 20 for granulocytes with Entrez-based gene definitions for array annotation [62]. We normalized raw fluorescence intensities by applying quantile normalization and robust multi-array average background correction. A quality assessment of the microarray data using the R/Bioconductor package arrayQualityMetrics [63] is provided as Supplementary Results. Raw and normalized microarray data have been deposited in NCBI’s Gene Expression Omnibus [64] and are accessible through GEO Series accession number GSE123731 (https://www.ncbi.nlm.nih.gov/geo/query/acc.cgi?acc= GSE123731).

For discovering reference genes in NK cells and granulocytes each, differential gene expression across the three discovery set patient groups (presurgical, SIRS, and sepsis) was first tested by one-way analysis of variance (ANOVA) with JMP® 10 Genomics version 6 (SAS Institute, Cary, NC, USA). Genes with a false-positive rate of α > 0.05 with false discovery rate correction for all three group pairings were considered nondiscriminatory. Among these, we selected the 5000 genes with the lowest variability across all samples from the three groups together using the standard deviation as an estimator [65]. Next, mean normalized log_2_ signal intensities for all three groups were required to be between 4 and 11 to avoid selecting genes with very low and very high expression levels, respectively. For granulocytes, this yielded a shortlist of only 18 genes, while 4831 genes remained for NK cells. Starting with the lowest variability, we sought to identify from each of these lists five genes with *p*-values > 0.5 from unadjusted t-tests for all three patient group pairings. 

### 4.6. RT-PCR

Total RNA was reverse-transcribed using the high capacity cDNA reverse transcription kit (Applied Biosystems, Thermo Fisher). TaqManTM RT-PCR gene expression assays (summarized in the Appendix A, Table A1) were performed on a 7900HT Fast Real Time PCR instrument (Applied Biosystems). Assays were run in triplicate on 96-well plates. Sixteen known reference genes were screened on 384-well micro fluidic cards with the TaqMan Endogenous Control Card (Applied Biosystems, catalog number 4367563). Data are represented as Ct values which are inversely proportional to target cDNA copy numbers in the PCR reactions, i.e., to transcript quantities. Calculations of fold differences were based on equal amounts of total RNA employed in each reverse transcription reaction.

### 4.7. Multiplex Gene Expression Assay

QGP-branched DNA signal amplification assays (Table A1) were run on a magnetic bead array platform (MAGPIX®, Luminex Corporation, Austin, TX, United States). Freshly isolated validation set NK cells and granulocytes were lysed according to the manufacturer’s protocol for PBMCs at 10^3^ cells/µL (Affymetrix, QuantiGene Sample Processing Kit, Cultured Cells). Eighty microliters of lysate were subjected in duplicates to hybridization and signal amplification. Data were analyzed following the QGP Assay manual and are represented as background corrected MFI values. Calculations of fold differences were based on equal numbers of lysed cells employed in each hybridization and signal amplification reaction.

### 4.8. External Transcriptome Dataset Retrieval

*AKIRIN1* expression profiles in curated GEO DataSets were retrieved from the GEO Profiles database [66] available online at www.ncbi.nlm.nih.gov/geoprofiles (accessed on October 22 2018). The search string “((homo sapiens[Organism]) AND neutrophil[Sample Source]) AND AKIRIN1” returned four records (Table 3). Mean fold differences in *AKIRIN1* expression levels and *p*-values from unadjusted *t*-tests were calculated for pairwise comparisons of the sample groups defined within each of these records.

### 4.9. Statistical Analyses

We used the Kruskal-Wallis test with Dunn’s test for post-hoc pairwise comparisons to calculate differences in patient characteristics in the three discovery set groups. Differences in patient characteristics and gene expression in pairs of patient groups were calculated using the Wilcoxon rank-sum test. Group differences for paired samples from in vitro experiments were evaluated using the Wilcoxon signed-rank test. Tests were conducted with Prism 7 (GraphPad Software, San Diego, United States). *p*-values < 0.05 were considered statistically significant.

## Figures and Tables

**Table 1:** Patient characteristics for NK cells and granulocytes for the discovery set ^1^.

**NK cells**			
Patients contributing eligible NK cell preparations/enrolled patients	Presurgical (*n* = 19/20)	SIRS (*n* = 16/19)	Sepsis (*n* = 10/20)
Patient characteristics			
Age (years)	63.6 ± 12.3	61.8 ± 17.4	61.8 ± 17.6
Sex (Male/Female)	10/9	12/4	5/5
SOFA score	na ^2^	5.3 ± 2.3	13.1 ± 3.7 ****
CRP (mg/L)	na ^3^	80 ± 49 ^4^	213 ± 105 ***
Lactate (mmol/L)	na ^2^	1.8 ± 1.3 ^5^	4.1 ± 2.2 ^6^ **
WBC (cells/nL)	7.0 ± 1.9 ^7^	12.5 ± 4.8 ***	14.3 ± 9.1 *
*SIRS etiology*			
Abdominal surgery	N.A.^8^	*n* = 9	N.A.
Thoracic surgery	N.A.	*n* = 3	N.A.
Polytrauma	N.A.	*n* = 3	N.A.
Lumbar spinal stabilization	N.A.	*n* = 1	N.A.
*Septic focus*			
Abdominal	N.A.	N.A.	*n* = 5
Pulmonal	N.A.	N.A.	*n* = 5
**Granulocytes**			
Patients contributing eligible granulocyte preparations/enrolled patients	Presurgical (*n* = 11/20)	SIRS (*n* = 16/19)	Sepsis (*n* = 15/20)
Patient characteristics			
Age (years)	60.6 ± 12.9	63.1 ± 17.6	62.5 ± 17.6
Sex (Male/Female)	5/6	12/4	7/8
SOFA score	na ^2^	5.1 ± 2.4	12.7 ± 3.2 ****
CRP (mg/L)	na ^3^	75 ± 48 ^9^	226 ± 101 ****
Lactate (mmol/L)	na ^2^	2.0 ± 1.9 ^10^	3.5 ± 2.1 ^11^ **
WBC (cells/nL)	7.6 ± 2.4 ^7^	11.8 ± 4.6	16.6 ± 11.6 *
*SIRS etiology*			
Abdominal surgery	N.A.	*n* = 10	N.A.
Thoracic surgery	N.A.	*n* = 1	N.A.
Polytrauma	N.A.	*n* = 4	N.A.
Lumbar spinal stabilization	N.A.	*n* = 1	N.A.
*Septic focus*			
Abdominal	N.A.	N.A.	*n* = 11
Pulmonal	N.A.	N.A.	*n* = 4
Patients contributing both NK cells and granulocytes/enrolled patients	*n* = 10/20	*n* = 14/19	*n* = 9/20

^1^ Data are represented as mean ± standard deviation (sd); ^2^ na, not available; ^3^ For the majority of presurgical patients, CRP determination was not available or was under the limit of detection (<2.9 mg/L); ^4^ Mean and sd values are based on 13 out of 16 CRP determinations because three were under the limit of detection (<2.9 mg/L); ^5^ For three out of the 16 SIRS patients, lactate levels were above 2 mmol/L. For one patient in the same group, lactate levels were not available; ^6^ For two out of the 10 sepsis patients, lactate levels were below 2 mmol/L; ^7^ For two out of the 19 presurgical patients, WBC determinations were not available; ^8^ Not Applicable; ^9^ Mean and sd values are based on 12 out of 16 CRP determinations because four were under the limit of detection (< 2.9 mg/L); ^10^ For one out of the 16 SIRS patients, lactate levels were above 2 mmol/L; ^11^ For four out of the 15 sepsis patients, lactate levels were below 2 mmol/L; *** *p* < 0.001, **** *p* < 0.0001 from Wilcoxon rank-sum test for pairwise comparisons. *** *p* < 0.001 for presurgical versus SIRS from the Dunn’s test for post hoc pairwise comparisons after Kruskal-Wallis test. ** *p* < 0.01 for SIRS versus sepsis comparisons of lactate levels after Wilcoxon rank-sum test. * *p* < 0.05 for presurgical versus sepsis from the Dunn’s test for post hoc pairwise comparisons after Kruskal-Wallis test.

**Table 2:** Patient characteristics for NK cells and granulocytes for the validation set ^1^.

**NK cells**		
Patients contributing eligible granulocyte preparations/enrolled patients	SIRS (*n* = 18/22)	Sepsis (*n* = 15/23)
Patient characteristics		
Age (years)	68.7 ± 15.0	71.5 ± 11.8
Sex (Male/Female)	13/5	7/8
SOFA score	6.3 ± 2.7	10.4 ± 2.0 ****
CRP (mg/L)	59 ± 38 ^2^	323 ± 111 ****
Lactate (mmol/L)	1.6 ± 1.3 ^3^	2.3 ± 1.7 ^4^
WBC (cells/nL)	16.7 ± 5.9	18.6 ± 4.9
*SIRS etiology*		
Abdominal surgery	*n* = 7	N.A.
Thoracic surgery	*n* = 4	N.A.
Vascular surgery	*n* = 2	N.A.
Polytrauma	*n* = 4	N.A.
Otolaryngology surgery	*n* = 1	N.A.
*Septic focus*		
Abdominal	N.A.	*n* = 7
Pulmonal	N.A.	*n* = 5
Soft tissue	N.A.	*n* = 2
Urogenital	N.A.	*n* = 1
**Granulocytes**		
	SIRS (*n* = 22/22)	Sepsis (*n* = 18/23)
Patient characteristics		
Age (years)	66.3 ± 14.9	69.4 ± 13.3
Sex (Male/Female)	16/17	9/9
SOFA score	6.5 ± 2.9	10.8 ± 2.7 ****
CRP (mg/L)	59 ± 47 ^2^	313 ± 123 ****
Lactate (mmol/L)	1.9 ± 2.0 ^5^	2.9 ± 2.4 ^6^ *
WBC (cells/nL)	16.7 ± 6.4	15.3 ± 5.9
*SIRS etiology*		
Abdominal surgery	*n* = 8	N.A.
Thoracic surgery	*n* = 4	N.A.
Vascular surgery	*n* = 3	N.A.
Polytrauma	*n* = 6	N.A.
Otolaryngology surgery	*n* = 1	N.A.
*Septic focus*		
Abdominal	N.A.	*n* = 8
Pulmonal	N.A.	*n* = 8
Soft tissue	N.A.	*n* = 1
Urogenital	N.A.	*n* = 1
Patients contributing both NK cells and granulocytes/enrolled patients	*n* = 18/22	*n* = 15/23

^1^ Data are represented as mean ± standard deviation; ^2^ For one patient, who contributed both cell types, CRP determination was under the limit of detection (< 2.9 mg/L); ^3^ For one out of 11 available lactate determinations, lactate levels were above 2 mmol/L; ^4^ For nine out of the 15 sepsis patients, lactate levels were below 2 mmol/L; ^5^ For three out of 15 available lactate determinations, lactate levels were above 2 mmol/L; ^6^ For ten out of the 18 sepsis patients, lactate levels were below 2 mmol/L; **** *p* < 0.0001, * *p* < 0.05 from Wilcoxon rank-sum test.

**Table 3:** Mean fold differences in *AKIRIN1* expression levels and *p-*values for group comparisons in neutrophil transcriptome datasets identified from GEO Profiles.

GEO Profile ID ^1^	Comparison Groups (*n*)			Mean Fold Differences ^3^			Publication
		*p*-Values ^2^					
48169967	Group number	1	2	3	4		Tang et al., 2008 [32]
	1 Controls (17)		1.04	0.93	0.79		
	2 Mixed infection sepsis (12)	0.76		0.89	0.76		
	3 Gram-negative sepsis (25)	0.54	0.35		0.85		
	4 Gram-positive sepsis (18)	0.12	0.08	0.23			
41143967	Group number	1	2	3	4		Tang et al., 2007 [31]
	1 Controls training set (13)		0.80	0.82	0.81		
	2 Controls validation set (8)	0.15		1.02	1.02		
	3 Sepsis training set (26)	0.09	0.86		1.00		
	4 Sepsis validation set (38)	0.07	0.89	0.96			
28190858	Group number	1	2	3			Silva et al., 2007 [33]
	1 In vitro controls (8)		0.91	0.99			
	2 In vitro LPS treated (8)	0.39		1.09			
	3 In vitro HMGB1 treated (8)	0.94	0.51				
29128858	Group number	1	2	3	4	5	Coldren et al., 2006 [34]
	1 In vitro controls (5)		0.83	0.40	0.38	0.36	
	2 In vitro LPS (5)	0.33		0.49	0.46	0.44	
	3 Circulating pre-LPS (12)	<10^−3^	0.04		0.94	0.90	
	4 Circulating post-LPS (14)	<10^−3^	0.03	0.52		0.96	
	5 Alveolar post-LPS (15)	<10^−3^	0.03	0.49	0.77		

^1^ Data were retrieved from the GEO Profiles database accessible at www.ncbi.nlm.nih.gov/geoprofiles; ^2^*p*-values are from unadjusted *t*-tests. ^3^ In the calculation of mean fold differences between groups, the mean expression value for the group higher on the list in the comparison group column (lower group number) was always the denominator and the value for the group lower on the list (higher group number) the numerator.

## Supplementary Materials

Supplementary materials can be found at https://www.mdpi.com/1422-0067/20/9/2290/s1; Supplementary Results (Supplementary Results.pdf) arrayQualityMetrics reports for SIRS and septic shock NK cells and granulocytes; Supplementary Tables (Supplementary Tables.pdf); Supplementary Table S1: Demographic and clinical patient characteristics; Supplementary Table S2: Specifications for monoclonal antibody-fluorochrome conjugates and their combinations used in flow cytometry.

## Abbreviations

*ADRBK1*	G protein-coupled receptor kinase 2
ALI	Acute lung injury
CRP	C-reactive protein
Ct	Threshold cycle
*DNAJA2*	DnaJ heat shock protein family (Hsp40) member A2
FBS	Fetal bovine serum
*GAPDH*	Glyceraldehyde-3-phosphate dehydrogenase
*GUSB*	Glucuronidase beta
HMGB1	High mobility group box 1 protein
*HPRT1*	Hypoxanthine phosphoribosyltransferase 1
ICU	Intensive care unit
IL-15	Interleukin-15
*INSM1*	INSM transcriptional repressor 1
LPS	Lipopolysaccharide
*LY6G6E* NK	Lymphocyte antigen 6 family member G6E Natural killer
PBMCs	Peripheral blood mononuclear cells
PBS	Phosphate buffered saline
*POLR2A*	RNA polymerase II subunit A
*PPIB*	Peptidylprolyl isomerase B
*PPP6R3*	Protein phosphatase 6 regulatory subunit 3
*P4HA1*	Prolyl 4-hydroxylase subunit alpha 1
QGP	QuantiGene Plex
*RAB2A*	RAB2A, member RAS oncogene family
sd	Standard deviation
SIRS	Systemic inflammatory response syndrome
SOFA	Sequential organ failure assessment score
*TAX1BP1*	Tax1 binding protein 1
*TNF*	Tumor necrosis factor
*YTHDF3*	YTH N6-methyladenosine RNA binding protein 3
WBCs	White blood cell counts

## Appendix A

Table A1 summarizes the discovered candidate reference genes for NK cells and granulocytes together with known endogenous reference genes used in further comparisons.

**Table A1:** Gene information and specifications for the individual TaqMan (Applied Biosciences) and QGP (Affymetrix) assays used.

Full Name	Gene Symbol	Chromosomal Location	Taqman Assay ID	GenBank Accession Number ^1^
Candidate reference genes identified from microarray for NK cells				
G protein-coupled receptor kinase 2 ^2^	*ADRBK1*	11q13.2	Hs00176395_m1	NM_001619
akirin1	*AKIRIN1*	1p34.3	Hs01047800_g1	NM_024595
protein phosphatase 6 regulatory subunit 3	*PPP6R3*	11q13.2	Hs00217759_m1	NM_018312
Tax1 binding protein 1	*TAX1BP1*	7p15.2	Hs00195718_m1	NM_006024
DnaJ heat shock protein family (Hsp40) member A2	*DNAJA2*	16q11.2	Hs00195365_m1	NM_005880
Candidate reference genes identified from microarray for granulocytes				
RAB2A, member RAS oncogene family	*RAB2A*	8q12.1-q12.2	na ^3^	NM_002865
INSM transcriptional repressor 1	*INSM1*	20p11.23	na	NM_002196
YTH N6-methyladenosine RNA binding protein 3	*YTHDF3*	8q12.3	na	NM_152758
lymphocyte antigen 6 family member G6E	*LY6G6E*	6p21.33	na	NR_003673
Known endogenous reference genes				
glyceraldehyde-3-phosphate dehydrogenase	*GAPDH*	12p13.31	na	NM_002046
glucuronidase beta	*GUSB*	7q11.21	Hs00939627_m1	NM_000181
hypoxanthine phosphoribosyltransferase 1	*HPRT1*	Xq26.2-q26.3	na	NM_000194
RNA polymerase II subunit A	*POLR2A*	17p13.1	Hs00172187_m1	na
peptidylprolyl isomerase B	*PPIB*	15q22.31	Hs00168719_m1	NM_000942
Control genes for in vitro cell stimulation				
prolyl 4-hydroxylase subunit alpha 1	*P4HA1*	10q22.1	Hs00914594_m1	na
tumor necrosis factor	*TNF*	6p21.33	Hs00174128_m1	na

^1^ GenBank accession numbers identify QGP assays; ^2^ At the time of submission, the new symbol for the G protein-coupled receptor kinase 2 gene in the NCBI Entrez database was *GRK2*. However, the Entrez-based gene definitions for array annotation employed for NK cells used its alias *ADRBK1* which was retained unchanged in this work; ^3^ na, not applicable.
