# Supplementary material for: AKIRIN1: A Potential New Reference Gene in Human Natural Killer Cells and Granulocytes in Sepsis
Source: Int J Mol Sci. 2019 May 9;20(9):2290. doi: 10.3390/ijms20092290 (PMC6539838; doi:10.3390/ijms20092290)
Supplement: Supplementary file 1 [file ijms-20-02290-s001.pdf]

SUPPLEMENTARY TABLES

Supplementary Table S1 summarizes demographic and clinical characteristics of critically ill patients enrolled in this study.

Supplementary Table S2 identifies the fluorochromes and conjugated monoclonal antibodies used in flow cytometry. MACS isolated cells were analyzed with panels 1 and 2 in discovery set samples. For validation set and healthy donor cell samples, these were combined into panel 3.

Table S1. Demographic and clinical patient characteristics.

	**Discovery** (August 2012 – April 2014)		
	Presurgical (n = 20)	SIRS (n = 19)	Sepsis (n = 20)
Age (years)[1]	63.7 ± 12.0	61.8 ± 16.7	61.8 ± 16.7
Sex (Male/Female)	10/10	14/5	10/10
SOFA[2] score[1]	na[3]	5.1 ± 2.5	13.3 ± 3.1****
Lactate (mmol/L)[1]	na	2.2 ± 1.9[4]	3.2 ± 2.0
ICU length of stay (days)[1]	na	8.6 ± 11.8	13.3 ± 8.6
ICU mortality (n)	na	0	6

	**Validation** (September 2016 – April 2017)	
	SIRS (n = 22)	Sepsis (n = 23)
Age (years)	66.3 ± 14.9	67.7 ± 14.6
Sex (Male/Female)	16/6	10/13
SOFA score	6.5 ± 2.9	10.9 ± 2.5****
Lactate (mmol/L)	1.9 ± 2.0[5]	2.9 ± 2.3*
ICU length of stay (days)	4.2 ± 4.9	14.7 ± 15.1
ICU mortality (n)	0	7

[1] Data are represented as mean ± standard deviation.
[2] SOFA score, sequential organ failure assessment score
[3] na, not available
[4] For one out of the 19 SIRS patients, Lactate levels were not available.
[5] For six out of the 22 SIRS patients, Lactate levels were not available.
* $p < 0.05$ from Wilcoxon rank-sum test, **** $p < 0.0001$ from Wilcoxon rank-sum test.

Table S2. Specifications for monoclonal antibody-fluorochrome conjugates and their combinations used in flow cytometry.

**Antigen**	**Clone**	**Fluorochrome**
	Panel 1	
CD14	MφP9	FITC
CD45	2D1	PerCP
CD15	H198	APC
	Panel 2	
CD14	MφP9	FITC
CD56	NCAM 16.2	PE
CD3	SK7	PerCP
CD19	SJ25C1	APC-Cy7
	Panel 3	
CD3	UCHT1	FITC
CD56	NCAM 16.2	PE
CD45	2D1	PerCP
CD15	H198	APC
CD19	SJ25C1	APC-Cy7
CD16	B73.1	PE-Cy7
CD14	MφP9	BV421
